# Primary Sublingual S100-Positive Undifferentiated Pleomorphic Sarcoma in a Cat: A Case Report with Comprehensive Exclusionary Immunohistochemistry

**DOI:** 10.3390/vetsci13101036

**Published:** 2026-09-30

**Authors:** Yoo Suk Kim, Hwi-Yool Kim, Kwangsik Jang

**Affiliations:** 1 Gonggam Animal Medical Center, Gwangju 62225, Republic of Korea; yookim83@gmail.com; 2 Department of Veterinary Surgery, College of Veterinary Medicine, Konkuk University, Seoul 05029, Republic of Korea

**Keywords:** exclusionary immunohistochemistry, feline, S100-positive undifferentiated pleomorphic sarcoma, sublingual tumor, surgical extirpation

## Abstract

Undifferentiated pleomorphic sarcoma (UPS) is an exceptionally rare mesenchymal neoplasm in the feline oral cavity, presenting significant diagnostic and therapeutic challenges. A 10-year and 10-month-old, castrated male Korean Shorthair cat presented with a progressively enlarging sublingual mass measuring 2.77 cm × 3.02 cm × 1.86 cm. Computed tomography revealed a well-circumscribed, non-osteolytic sublingual mass without detectable mandibular bone erosion or regional lymphadenopathy. Gross complete surgical excision was performed via a transoral approach without mandibulectomy. Histopathological examination revealed a highly pleomorphic, sheet-like proliferation of atypical spindle to epithelioid cells with prominent giant binucleated cells and a low mitotic count (3 per 10 high-power fields). To establish a diagnosis of exclusion, an extensive immunohistochemical (IHC) panel was performed. The neoplastic cells were strongly positive for vimentin and diffusely positive for S100, but immunonegative for pancytokeratin (PanCK), Melan-A, Iba-1, desmin, and α-smooth muscle actin (α-SMA). These exclusionary IHC findings did not support epithelial, histiocytic, or myogenic differentiation, though amelanotic melanoma cannot be completely excluded based on Melan-A negativity alone. Rather than confirming definitive neural differentiation, the diffuse S100 immunoreactivity supported a final diagnosis of an S100-positive UPS. The patient achieved rapid functional recovery, with owner-reported absence of clinical signs of local recurrence over a 90-day follow-up period. This report underscores the necessity of a broad exclusionary IHC panel to accurately diagnose feline oral UPS and highlights surgical resection as a viable local management option.

## Simple Summary

A 10-year and 10-month-old neutered male Korean Shorthair cat presented with a progressively enlarging sublingual mass causing tongue displacement, ptyalism, and intermittent oral bleeding. Preoperative computed tomography (CT) revealed a well-circumscribed, heterogeneously enhancing soft tissue mass in the left sublingual space without CT evidence of osteolysis or regional lymph node metastasis. Gross complete surgical excision via careful blunt dissection was achieved without intraoperative complications. Comprehensive histopathological examination with H&E staining demonstrated severe cellular pleomorphism, nuclear atypia, and occasional binucleated cells. An expanded immunohistochemical (IHC) panel was performed to establish a diagnosis of exclusion; the tumor cells exhibited strong, diffuse positivity for Vimentin and S100, while remaining entirely immunonegative for pancytokeratin, Melan-A, Iba-1, α-smooth muscle actin, and desmin. Based on these exclusionary IHC findings and S100 expression, a diagnosis of feline sublingual S100-positive undifferentiated pleomorphic sarcoma (UPS) was supported. Postoperative recovery was uneventful, with complete mucosal healing and total resolution of lingual displacement. At the 3-month follow-up, owner-reported absence of visible mass regrowth or recurrence-associated clinical signs was documented. This case highlights the surgical feasibility and critical diagnostic utility of an expanded exclusionary IHC panel in managing rare feline sublingual UPS.

## 1. Introduction

Feline soft tissue sarcomas (STSs) encompass a heterogeneous group of malignant mesenchymal neoplasms, with fibrosarcoma representing the most prevalent subtype in the oral cavity. Feline oral malignancies are predominantly aggressive squamous cell carcinomas, which can cause local tissue infiltration and bone lysis, particularly in advanced stages [1,2]. Undifferentiated pleomorphic sarcoma (UPS)—historically designated as malignant fibrous histiocytoma—is an exceptionally rare entity in cats, particularly within the lingual and sublingual regions [1,3]. Diagnostically, UPS represents a true diagnosis of exclusion [4,5]. Because its cytomorphological features—characterized by marked cellular pleomorphism, multinucleated giant cells, and bizarre mitotic figures—overlap significantly with poorly differentiated carcinomas, amelanotic melanomas, histiocytic sarcomas, and other specific sarcoma lineages, morphological evaluation alone is insufficient for a definitive diagnosis [3,4,6].

To establish an accurate diagnosis of UPS, a comprehensive immunohistochemical (IHC) panel is essential to systematically exclude alternative lines of differentiation [4,5,6]. While myogenic markers such as desmin and α-smooth muscle actin (α-SMA) are routinely employed, ruling out epithelial (pancytokeratin), melanocytic/neural (S100 calcium-binding protein [S100], Melan-A), and histiocytic (ionized calcium-binding adaptor molecule 1 [Iba-1]) lineages is mandatory [3,4,5,6].

In the feline oral cavity, sublingual masses present formidable surgical and diagnostic challenges due to restricted anatomical access, proximity to major neurovascular structures, and potential compromise of tongue function. Complete surgical resection with wide margins remains the cornerstone of STS management; however, aggressive radical procedures such as mandibulectomy often carry substantial morbidity [7,8].

This report describes a rare case of a large feline sublingual UPS alongside an extensive exclusionary IHC workup highlighting the diagnostic approach and surgical considerations for this uncommon neoplasm. Given the inherently limited lineage specificity of S100 protein, which can be expressed in various neural, melanocytic, and mesenchymal neoplasms, the persistent S100 positivity is interpreted cautiously, supporting the classification of an S100-positive UPS.

## 2. Case Description

### 2.1. Clinical Presentation

**Figure 1:**
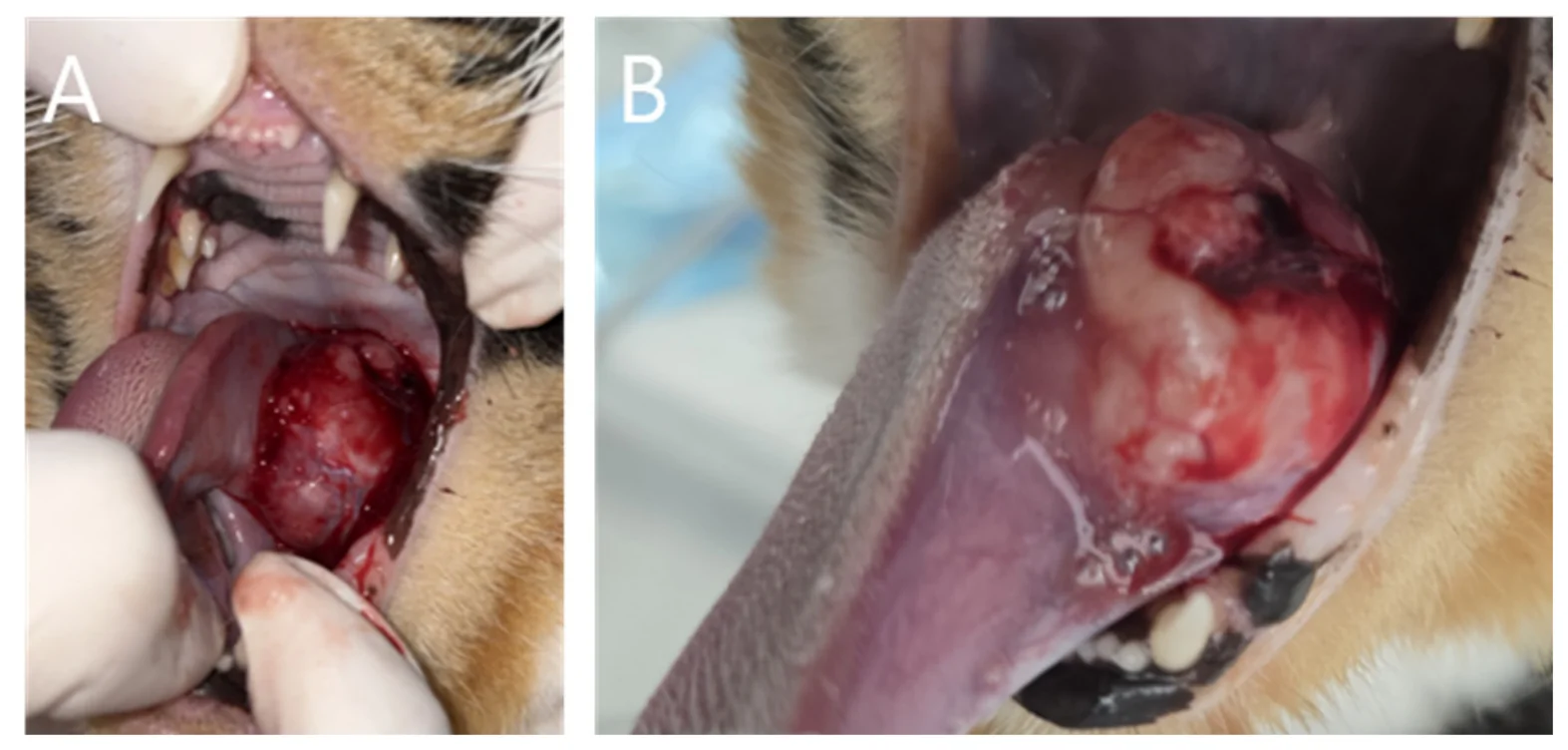
Macroscopic oral presentation of the patient at initial evaluation. (**A**) Gross appearance of the oral cavity displaying a large, lobulated nodular mass occupying the left sublingual space, accompanied by surface focal ulceration and hemorrhages. (**B**) Mechanical retraction of the tongue confirming the tumor origin within the sublingual space, demonstrating severe contralateral displacement of the lingual body.

A 10-year and 10-month-old neutered male Korean Shorthair cat presented with a progressively enlarging sublingual mass, severe ptyalism, and intermittent oral hemorrhage. The owner had noticed a small sublingual nodule approximately 3 months prior to presentation. Physical examination revealed a firm, well-demarcated nodular mass in the left sublingual space occupying more than half of the oral cavity (Figure 1A,B). Despite its size, the patient maintained voluntary solid food intake without severe dysphagia. Mandibular and retropharyngeal lymph nodes were within normal limits on palpation. General hematology and serum biochemistry revealed no clinically significant abnormalities. Preoperative fine-needle aspiration was withheld due to the risk of active hemorrhage from the highly vascularized mass [7].

### 2.2. Imaging Diagnosis

**Figure 2:**
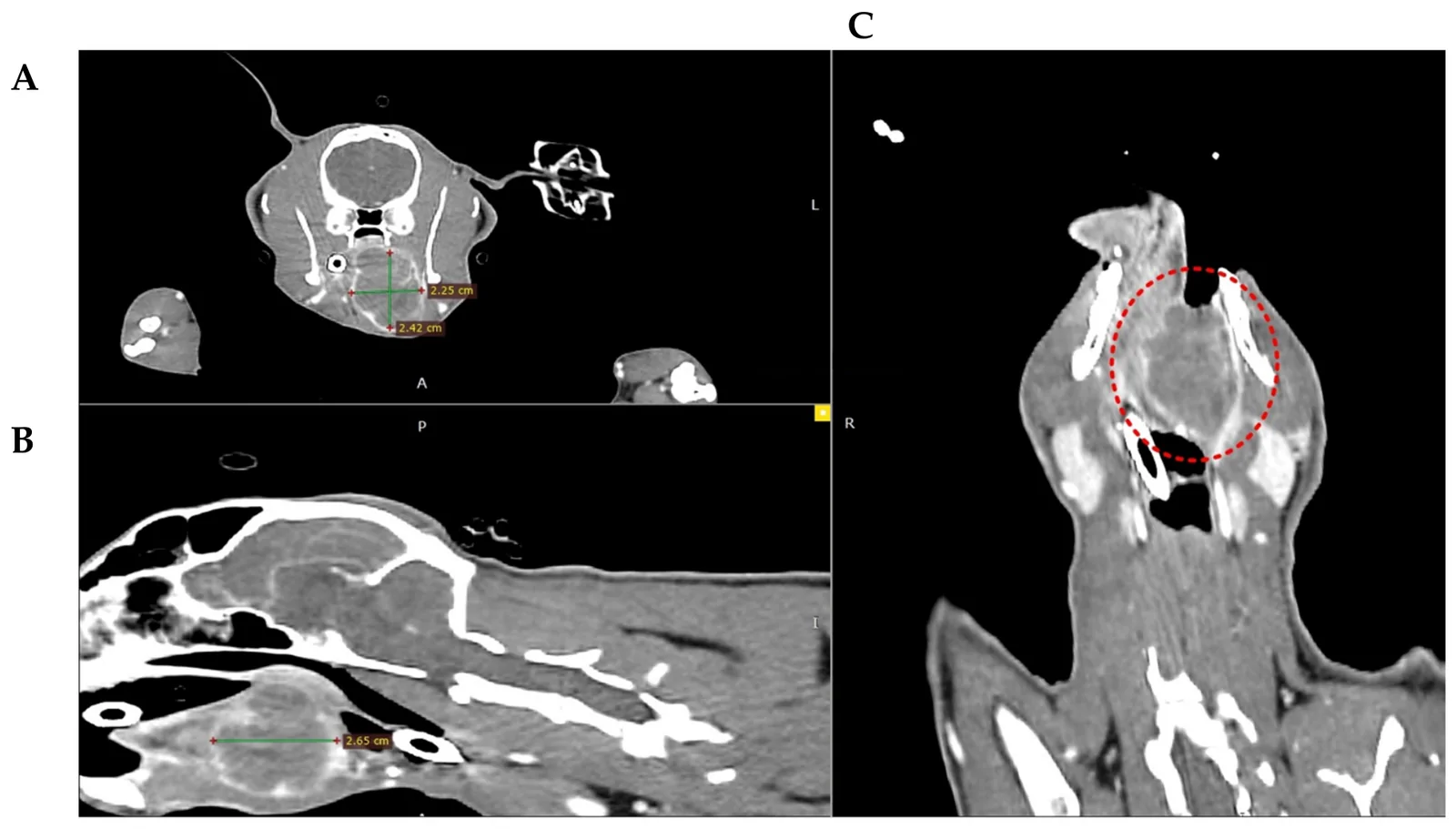
Contrast-enhanced computed tomography (CT) characteristics of the feline sublingual mass. (**A**) Transverse post-contrast CT image displaying a large, well-circumscribed, heterogeneously enhancing soft tissue mass within the left sublingual space, measuring 2.25 cm × 2.42 cm. (**B**) Sagittal reformatted image illustrating the longitudinal extent (2.65 cm) of the mass and dorsal displacement of the adjacent soft tissue structures. (**C**) Dorsal reformatted CT image (red dashed circle) demonstrating the expansive growth of the mass within the sublingual floor. Note the absence of cortical osteolysis, periosteal reaction, or aggressive structural destruction of the adjacent mandibles. A, anterior; P, posterior; R, right; L, left; I, inferior.

Computed tomography (CT) of the head was performed to evaluate the structural extent of the mass. Post-contrast CT images demonstrated a well-circumscribed, heterogeneously enhancing soft tissue mass measuring approximately 2.25 cm × 2.42 cm × 2.65 cm within the left sublingual space (Figure 2A,B). Peripheral rim enhancement and central hypoattenuation were noted, which may represent necrosis. Importantly, there was an absence of CT-detectable mandibular cortical osteolysis or periosteal reaction (Figure 2C). Parotid, mandibular, and medial retropharyngeal lymph nodes showed no definitive tomographic signs of regional metastasis, although occult microscopic invasion could not be completely excluded via standard CT alone [8]. Preoperative whole-body CT, including the thorax, demonstrated no evidence of pulmonary metastasis or thoracic lymphadenopathy.

### 2.3. Surgical Procedure and Anesthetic Management

Preanesthetic medications included omeprazole (1 mg/kg, IV), amoxicillin–clavulanic acid (12.5 mg/kg, IV), maropitant (1 mg/kg, IV), and butorphanol (0.2 mg/kg, IV). Anesthesia was induced with propofol (6 mg/kg, IV) and maintained with isoflurane in 100% oxygen following endotracheal intubation. For intraoperative analgesia and anesthetic sparing, a continuous rate infusion (CRI) combination of remifentanil (0.08–0.16 µg/kg/min), midazolam (0.01–0.02 mg/kg/h), and ketamine (0.14–0.28 mg/kg/h) in 0.9% normal saline was administered and titrated based on anesthetic depth and autonomic responses.

**Figure 3:**
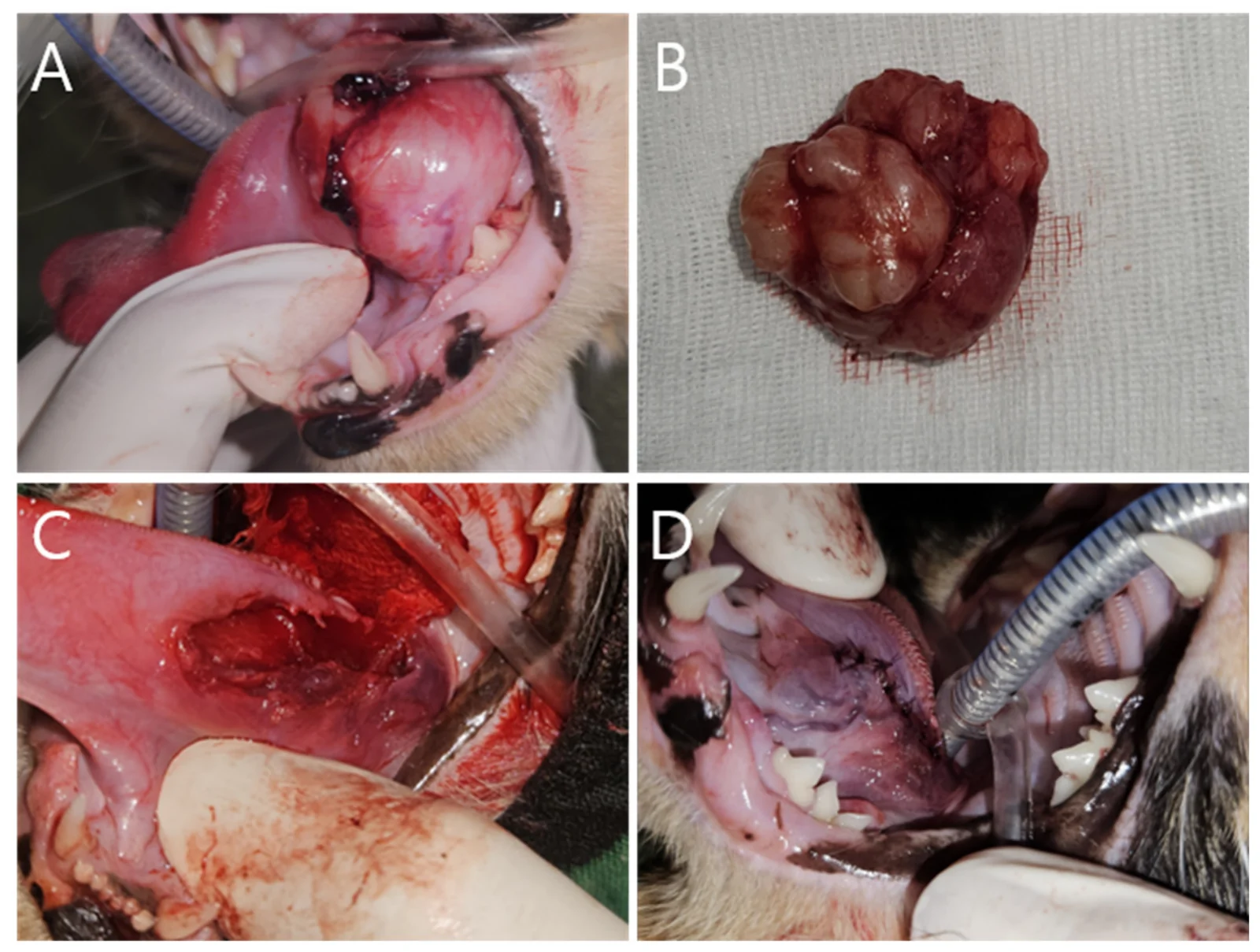
Intraoperative findings and step-by-step surgical extirpation of the sublingual mass. (**A**) Initial oral inspection under general anesthesia. Mechanical retraction of the tongue facilitates identification of the mass margins for the surgical incision. (**B**) Post-extirpation image of the sublingual mass in toto, displaying a distinct multinodular, lobulated morphology. (**C**) Intraoperative view of the surgical bed immediately following the mass removal. A clear plane of dissection is visible, allowing for blunt dissection and careful preservation of the adjacent deep lingual neurovascular bundles with minimal hemorrhage. (**D**) Primary closure and simple interrupted apposition of the sublingual mucosal defect using 5-0 poliglecaprone 25, restoring the anatomical alignment of the tongue.

With the patient in dorsal recumbency, a mucosal incision was made over the mass (Figure 3A). Gross complete surgical excision was achieved via careful blunt dissection along the fibrous pseudocapsule, preserving the adjacent left lingual artery, lingual nerve, and hypoglossal nerve (Figure 3B). The excised multilobulated mass measured 2.77 cm × 3.02 cm × 1.86 cm in gross dimensions (Figure 3C). The sublingual mucosal defect was closed with 5–0 poliglecaprone 25 in a simple interrupted pattern (Figure 3D).

### 2.4. Histopathological and Immunohistochemical Analyses

**Figure 4:**
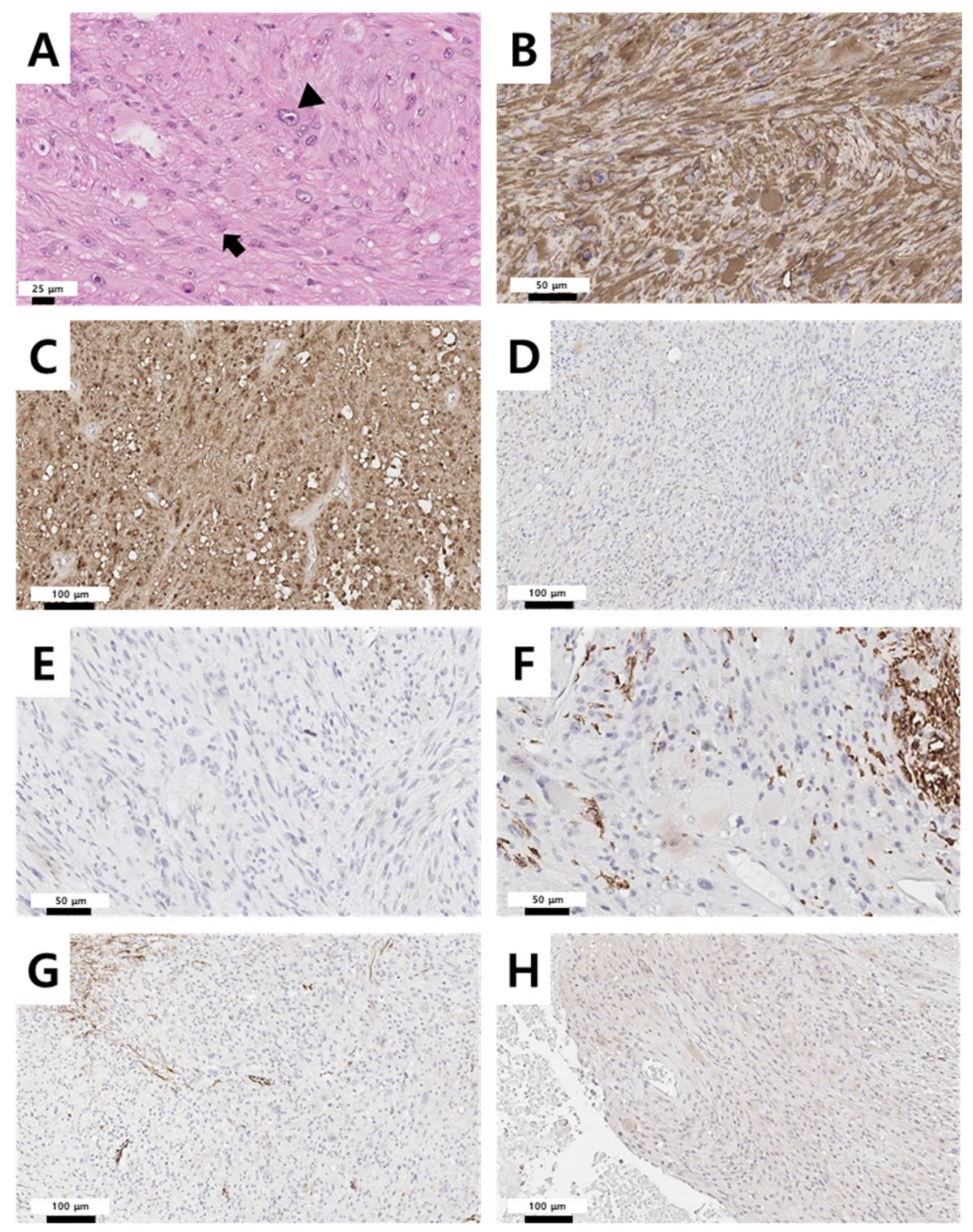
Comprehensive histopathological and immunohistochemical (IHC) profiling of the feline sublingual mass. (**A**) Hematoxylin and eosin (H&E) staining displaying dense, unorganized sheets of highly pleomorphic, malignant spindle-to-epithelioid cells with severe anisokaryosis and prominent multiple nucleoli. An atypical binucleated tumor cell (arrow) and severe cellular pleomorphism/nuclear atypia (arrowhead) are indicated (scale bar = 25 µm). (**B**) Strong and diffuse cytoplasmic immunoreactivity for Vimentin, confirming mesenchymal lineage (scale bar = 50 µm). (**C**) Diffuse cytoplasmic positivity for S100 calcium-binding protein (S100), demonstrating S100 immunoreactivity within the pleomorphic neoplastic population (scale bar = 100 µm). (**D**) Complete immunonegativity for pancytokeratin (PanCK), excluding carcinoma (scale bar = 100 µm). (**E**) Immunonegativity for Melan-A, which does not support melanocytic differentiation, although amelanotic melanoma cannot be definitively excluded based solely on negative Melan-A (scale bar = 50 µm). (**F**) Immunonegativity for ionized calcium-binding adaptor molecule 1 (Iba-1) within primary neoplastic cells, with resident stromal macrophages serving as internal positive controls, ruling out histiocytic sarcoma (scale bar = 50 µm). (**G**) Definitive cytoplasmic negativity for α-smooth muscle actin (α-SMA), with tunica media smooth muscle of native blood vessels serving as an internal positive control (scale bar = 100 µm). (**H**) Definitive cytoplasmic negativity for desmin, with adjacent non-neoplastic lingual skeletal muscle serving as the internal positive control, not supporting rhabdomyoblastic differentiation (scale bar = 100 µm).

Following complete surgical extirpation, the resected tissue specimens were fixed in 10% neutral buffered formalin and submitted to an external veterinary diagnostic laboratory (GREEN VET, Yongin-si, Republic of Korea) for definitive histopathological and immunohistochemical evaluation. Histopathological examination (H&E) revealed a dense, unorganized proliferation of highly pleomorphic spindle-to-epithelioid cells with severe anisokaryosis and prominent nucleoli (Figure 4A). Occasional binucleated cells were identified, whereas classic multinucleated giant cells were absent. The mitotic count was 3 per 10 high-power fields (3 per 2.37 mm^2^). Neoplastic cells extended close to the surgical resection lines.

To establish a diagnosis of exclusion, an expanded IHC panel was conducted. The neoplastic cells demonstrated strong, diffuse cytoplasmic reactivity for vimentin (Figure 4B) and diffuse positivity for S100 (Figure 4C). Conversely, neoplastic cells were completely immunonegative for pancytokeratin (PanCK) (Figure 4D), Melan-A (Figure 4E), Iba-1 (Figure 4F), α-SMA (Figure 4G), and desmin (Figure 4H). Internal positive controls (normal skeletal muscle, vascular smooth muscle, and stromal histiocytes) functioned appropriately. The comprehensive antibody panel, including target antigens, clone specifications, host species, working dilutions, antigen retrieval conditions, and manufacturer is summarized in Table 1. The complete absence of PanCK, Melan-A, Iba-1, α-SMA, and desmin ruled out epithelial (carcinoma/sarcomatoid SCC), melanocytic (melanoma), histiocytic, and myogenic (rhabdomyosarcoma/leiomyosarcoma) differentiation. Based on the exclusionary IHC findings combined with diffuse S100 expression, a final diagnosis of an S100-positive UPS was supported [3,4,5]. Immunohistochemistry for myogenic differentiation 1 (MyoD1) was also performed to further investigate rhabdomyoblastic differentiation; however, neoplastic cells exhibited only faint, diffuse, non-specific cytoplasmic background staining without demonstrable nuclear labeling. Given that diagnostically definitive MyoD1 reactivity requires nuclear expression, and cross-species antibody reactivity in felines may yield non-specific cytoplasmic artifact, this finding was interpreted as undetermined/negative and did not support skeletal muscle differentiation.

### 2.5. Postoperative Management and Follow-Up

**Figure 5:**
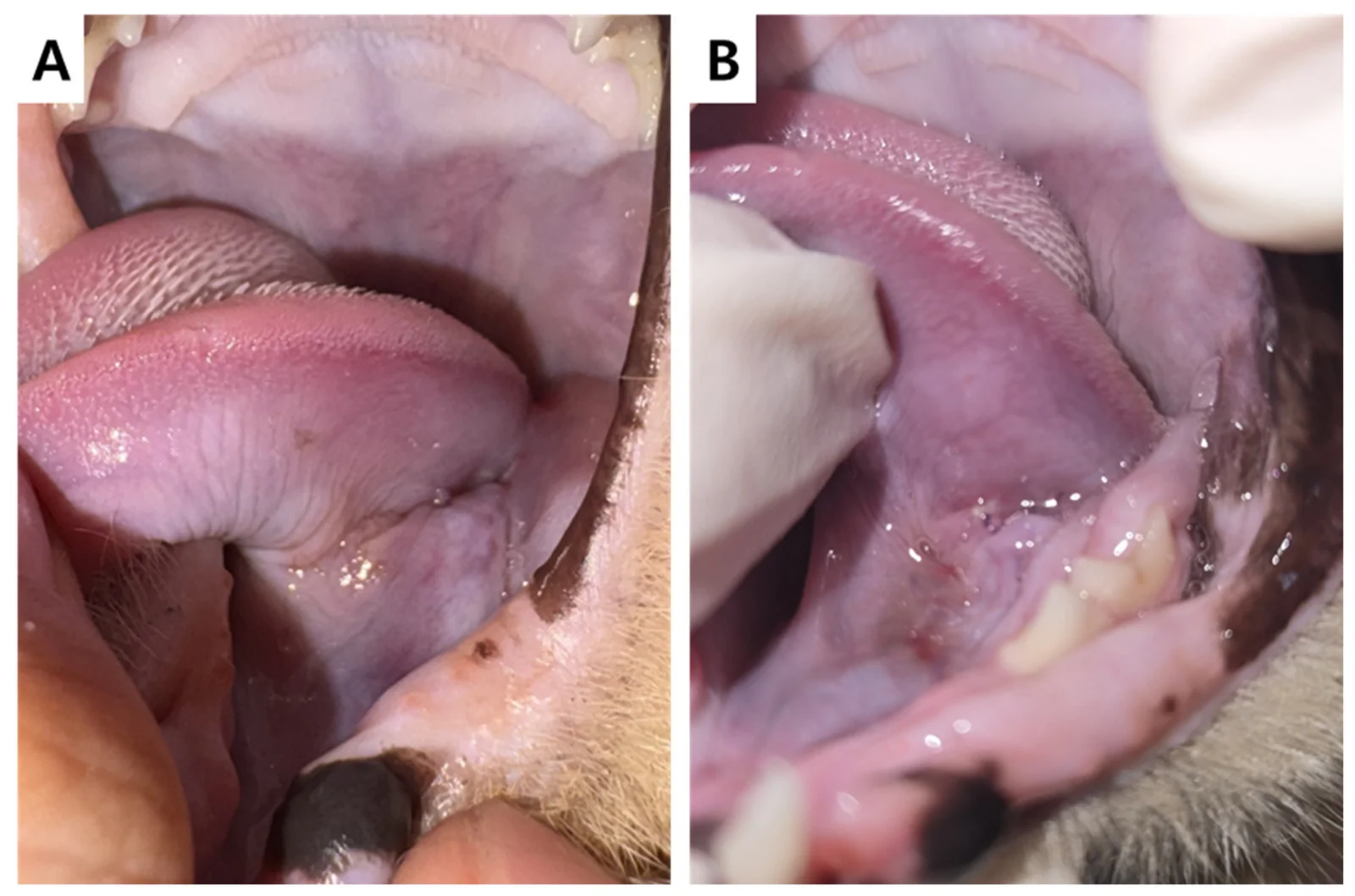
Postoperative intraoral clinical follow-up demonstrating mucosal healing. (**A**) Gross intraoral view at 1 day post-extirpation, showing mucosal adaptation and proper alignment of the tongue without acute surgical complications or active hemorrhage. (**B**) Gross intraoral view at the 1-week postoperative follow-up. The surgical bed shows progressive mucosal healing with smooth tissue continuity, complete re-epithelialization, and resolution of the previous lingual displacement.

Postoperative recovery was uneventful. For 7 days post-surgery, oral medications were administered as follows: amoxicillin–clavulanic acid (12.5 mg/kg, PO, q12h), gabapentin (5 mg/kg, PO, q12h), omeprazole (1 mg/kg, PO, q12h), and maropitant (1 mg/kg, PO, q24h). Voluntary intake of soft food resumed within 12 h post-surgery. Re-evaluations at post-operative day 1 and 1 week confirmed stable mucosal healing and normal lingual mobility (Figure 5A,B). At the 90-day (3-month) milestone, structured telephone follow-up with the owner indicated that the patient maintained a healthy general condition with normal prehension and appetite, without owner-observed swallowing difficulties or visible mass regrowth. Due to owner compliance constraints, in-person physical oral examination and follow-up diagnostic imaging for local recurrence or distant metastasis could not be performed.

## 3. Discussion

Oral cavity soft tissue sarcomas (STSs) in cats are aggressive mesenchymal neoplasms, with fibrosarcomas comprising the vast majority [1,2]. UPS—formerly classified under malignant fibrous histiocytoma—is extraordinarily rare in the feline oral cavity, particularly in the sublingual space [3]. Morphologically, UPS is defined by marked cellular pleomorphism, multinucleated giant cells, and variable mitotic activity. However, because these histomorphological features overlap substantially with poorly differentiated carcinomas, amelanotic melanomas, histiocytic sarcomas, and distinct myogenic or neural sarcomas, UPS remains a true diagnosis of exclusion [3,4,5,6].

To establish a definitive diagnosis, a comprehensive IHC panel is mandatory to exclude alternative lineages. In the present case, light microscopy alone was insufficient to determine the histiogenesis. The initial differential diagnosis included sarcomatoid squamous cell carcinoma, amelanotic melanoma, rhabdomyosarcoma, leiomyosarcoma, histiocytic sarcoma, and malignant peripheral nerve sheath tumor (PNST) [3,6,9].

Through an expanded exclusionary IHC panel, the neoplastic cells demonstrated strong, diffuse immunoreactivity for vimentin, confirming their mesenchymal origin, while remaining immunonegative for PanCK, Melan-A, Iba-1, desmin, and α-SMA. The complete negativity for PanCK and Iba-1 did not support epithelial (carcinoma) and histiocytic lineages, respectively [4,5,6]. Furthermore, although Melan-A negativity did not support melanocytic differentiation, amelanotic melanoma cannot be definitively ruled out based solely on this single marker [4,6]. Negativity for desmin and α-SMA did not support myogenic origin, making leiomyosarcoma and rhabdomyosarcoma less likely. Additionally, as reported in the results, MyoD1 demonstrated no true nuclear immunoreactivity; the faint, non-specific cytoplasmic background was consistent with cross-species antibody reactivity limitations in cats rather than evidence of skeletal muscle differentiation.

Intriguingly, the neoplastic cells exhibited diffuse cytoplasmic positivity for S100. S100 is a highly sensitive but non-specific marker expressed in neuroectodermal tissues, neural crest-derived tumors (e.g., PNST, melanoma), chondroid, and some myoepithelial neoplasms [4,10]. While S100 positivity might raise suspicion for an amelanotic melanoma, negative Melan-A immunoreactivity alone does not conclusively exclude melanocytic neoplasms. In a retrospective study of 48 feline melanomas, Ramos-Vara et al. [11] demonstrated that only 67% expressed Melan-A, and 9 of 12 amelanotic melanomas were completely immunonegative for Melan-A, with S100 remaining positive across many Melan-A-negative cases. Furthermore, while classic histopathological patterns characteristic of Schwannomas (such as Antoni A/B areas or Verocay bodies) were absent on routine H&E examination, the absence of these features does not exclude a high-grade or pleomorphic variant of malignant PNST. Additional lineage markers such as SOX10, GFAP, or PNL2 would be required to definitively confirm or exclude these neurogenic and melanocytic differential diagnoses. Therefore, rather than definitively asserting a neural origin or neural immunophenotype, or partial neurogenic lineage, this neoplasm is most appropriately designated as an S100-positive UPS.

From a therapeutic perspective, achieving complete surgical margins in the feline sublingual region is notoriously difficult due to dense neurovascular networks and limited anatomical room. While radical surgical approaches such as mandibulectomy are frequently advocated for oral STSs to obtain wide microscopic margins, they carry substantial postoperative morbidity [7,8]. In this case, a transoral gross complete excision preserving adjacent neurovascular structures and mandibular continuity provided immediate symptomatic relief and rapid restoration of tongue function. When managing soft tissue sarcomas with incomplete or narrow surgical margins, adjuvant therapies such as metronomic chemotherapy [12] or postoperative radiation therapy [13] have been investigated in veterinary medicine (largely extrapolated from canine studies) to reduce local recurrence. However, robust clinical evidence validating their long-term efficacy specifically in feline oral sarcomas remains scarce, and treatment responses in cats are highly variable.

This report has several methodological limitations that warrant consideration. First, while gross complete excision was accomplished, histopathological examination indicated close surgical margins. Second, although preoperative whole-body CT confirmed the absence of pulmonary metastasis or thoracic lymphadenopathy, post-operative imaging follow-up could not be performed due to owner compliance constraints. Finally, although structured telephone follow-up provided valuable subjective information regarding functional recovery and appetite at 90 days, remote follow-up alone cannot definitively exclude subclinical local recurrence or late distant metastasis, which ideally requires periodic sedation, physical oral examination, and follow-up imaging. Despite these limitations, the detailed diagnostic workup presented here highlights the critical necessity of an extended exclusionary IHC panel for accurate diagnosis and management of rare feline sublingual pleomorphic sarcomas.

## 4. Conclusions

In summary, this report describes an exceptionally rare case of feline sublingual S100-positive UPS. This case demonstrates that histomorphological evaluation alone is insufficient for diagnosing feline oral pleomorphic mesenchymal neoplasms. Establishing an accurate diagnosis of UPS necessitates an extensive exclusionary IHC panel—including PanCK, Melan-A, Iba-1, and myogenic markers—to systematically investigate alternative epithelial, melanocytic, histiocytic, and muscle-derived mimics. Furthermore, gross complete surgical excision via a transoral approach provided immediate functional recovery and local disease control without requiring extensive mandibulectomy, achieving an owner-reported absence of signs suggestive of recurrence over a 90-day follow-up period. Despite limitations in long-term imaging follow-up, our findings highlight the essential role of comprehensive IHC workups in feline oral oncological pathology and support conservative, function-sparing surgical resection as a viable local management option for sublingual sarcomas.

## Figures and Tables

**Table 1:** Panel of primary antibodies used for immunohistochemical evaluation.

Primary Antibody	Clone/Type	Host Species	Dilution	Antigen Retrieval	Manufacturer
Vimentin	V9	Mouse monoclonal	1:1000	Citric acid (pH 6.0), HIER	Dako (Agilent Technologies, Santa Clara, CA, USA)
S100	Polyclonal	Rabbit polyclonal	1:2000	Citric acid (pH 6.0), HIER	Dako
PanCK	AE1/AE3	Mouse monoclonal	1:200	Citric acid (pH 6.0), HIER	Dako
Melan-A	A103	Mouse monoclonal	1:100	Tris-EDTA (pH 9.0), HIER	Leica Biosystems (Newcastle upon Tyne, UK)
Iba-1	Polyclonal	Rabbit polyclonal	1:500	Citric acid (pH 6.0), HIER	FUJIFILM Wako Pure Chemical (Osaka, Japan)
α-SMA	1A4	Mouse monoclonal	1:200	Citric acid (pH 6.0), HIER	Dako
Desmin	D33	Mouse monoclonal	1:50	Citric acid (pH 6.0), HIER	Dako
MyoD1	5.8A	Mouse monoclonal	1:100	Citric acid (pH 6.0), HIER	Invitrogen (Thermo Fisher Scientific, Waltham, MA, USA)

α-SMA, α-smooth muscle actin; HIER, heat-induced epitope retrieval; Iba-1, ionized calcium-binding adaptor molecule 1; MyoD1, myogenic differentiation 1; PanCK, pancytokeratin; S100, S100 calcium-binding protein, S100.

## Data Availability

The original contributions presented in this study are included in the article. Further inquiries can be directed to the corresponding authors.

## Abbreviations

The following abbreviations are used in this manuscript:

AMC	Amoxicillin–clavulanic acid
α-SMA	α-smooth muscle actin
CT	Computed tomography
H&E	Hematoxylin and eosin
HPF	High-power field
Iba-1	Ionized calcium-binding adaptor molecule 1
IHC	Immunohistochemical/Immunohistochemistry
PanCK	Pancytokeratin
PNST	Peripheral nerve sheath tumor
S100	S100 calcium-binding protein
SCC	Squamous cell carcinoma
STS	Soft tissue sarcoma
UPS	Undifferentiated pleomorphic sarcoma
